# Tubulin posttranslational modifications modify the atypical spermatozoon centriole

**DOI:** 10.17912/micropub.biology.000678

**Published:** 2022-11-12

**Authors:** Katerina A. Turner, Derek F. Kluczynski, Ryan J. Hefner, Rami B. Moussa, Julia N. Slogar, Joice B. Thekkethottiyil, Haley D. Prine, Emily R. Crossley, Lucas J. Flanagan, Marlena M. LaBoy, Mia B. Moran, Taylor G. Boyd, Benjamin A. Kujawski, Kelsie Ruble, John M. Pap, Ankit Jaiswal, Tariq A. Shah, Puneet Sindhwani, Tomer Avidor-Reiss

**Affiliations:** 1 The University of Toledo, Toledo, Ohio, USA.

## Abstract

Sperm cells are transcriptionally and translationally silent. Therefore, they may use one of the remaining mechanisms to respond to stimuli in their environment, the post-translational modification of their proteins. Here we examined three post-translational modifications, acetylation, glutamylation, and glycylation of the protein tubulin in human and cattle sperm. Tubulin is the monomer that makes up microtubules, and microtubules constitute the core component of both the sperm centrioles and the axoneme. We found that the sperm of both species were labeled by antibodies against acetylated tubulin and glutamylated tubulin.

**Figure 1. Acetylated tubulin and glutamylated tubulin are observed in both cattle and human sperm centrioles, while glycylated tubulin staining is variable:**
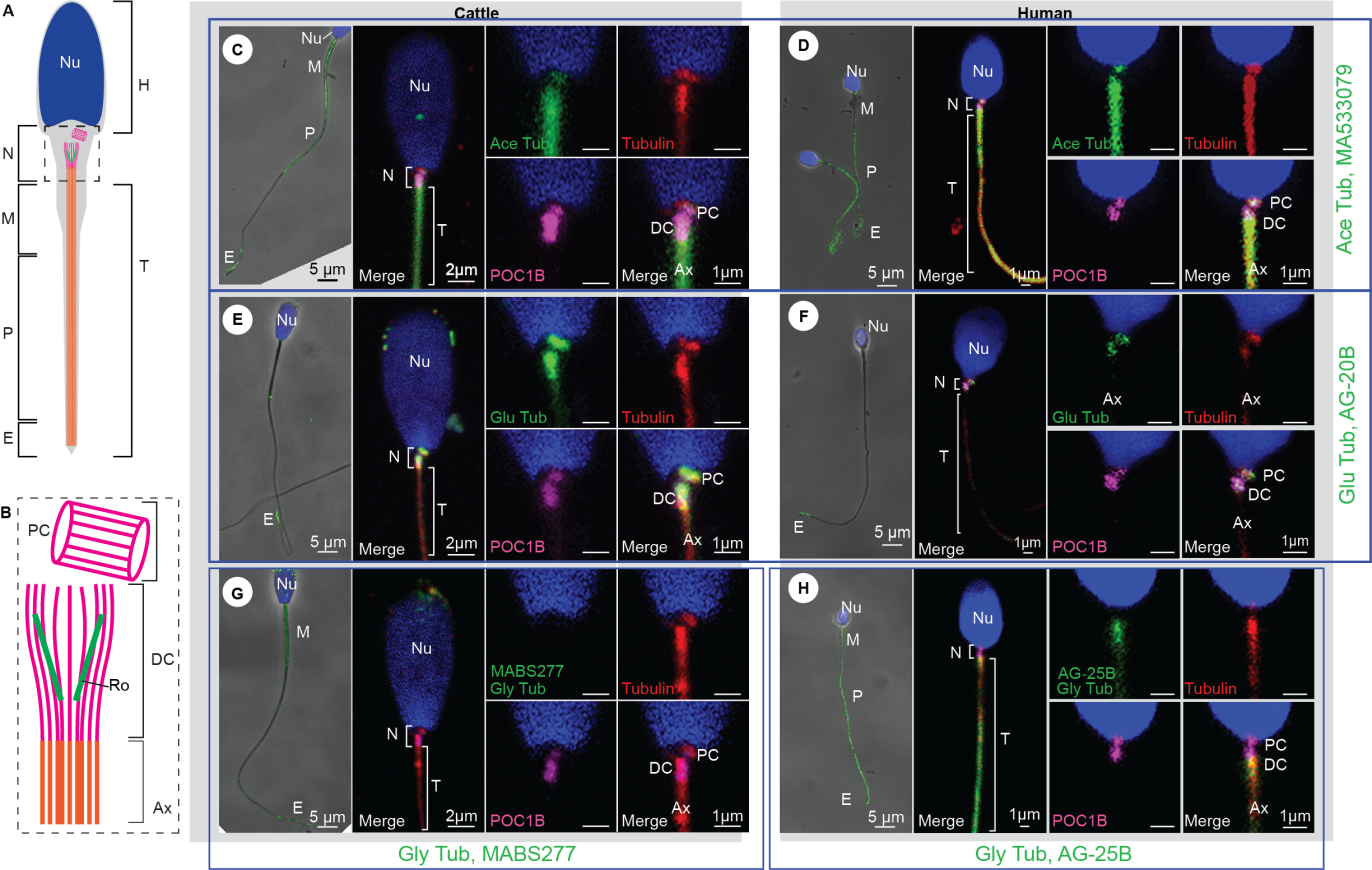
**A** ) The three main parts of cattle and human sperm are the head (H), neck (N), and tail (T). The head contains the nucleus (Nu), the proximal centriole (PC) and distal centriole (DC) are within the neck, and the tail consists of the axoneme (Ax), which extends through the midpiece (M), the principal piece (P), and end piece (E). **B** ) The proximal centriole is barrel-shaped, comprised of nine microtubule triplets, and is nearly perpendicular to the anterior side of the distal centriole. The distal centriole has an atypical structure with two protein rods (Ro), has nine splayed doublet microtubules, and is directly connected to the axoneme. **C-H** ) Immunofluorescence staining was used to observe different proteins and posttranslational modifications in cattle ( **C** , **E** , **G** ) and human ( **D** , **F** , **H** ) sperm. POC1B (magenta) is observed almost exclusively in the proximal and distal centrioles. Tubulin (red) labels the PC, DC, and axoneme. **C-D** ) The antibody (Thermo Fisher Scientific, MA533079) against acetylated tubulin (Ace Tub, green) was primarily detected in the PC, DC, and especially in the axoneme of cattle ( **C** ) and human ( **D** ) sperm. **E-F** ) The antibody (Adipogen, AG-20B-0020-C100) against glutamylated tubulin (Glu Tub, green) labeled the PC and DC of cattle ( **E** ) and human ( **F** ) sperm. **G** ) The antibody (EMD Millipore, MABS277) against glycylated tubulin (Gly Tub, green) was not detected in any centriolar components of cattle. **H** ) The antibody (Adipogen, rabbit ab, AG-25B-0034-C100) against monoglycylated tubulin (Gly Tub, green) was not detected in the PC of human sperm, but there is some signal in the midpiece and DC. Note that only the tail structures labeled by the anti-posttranslational modification antibody are annotated in the low magnification panels.

## Description

Infertility affects 12-15% of couples; furthermore, one-third of couples have unexplained infertility (Thoma *et al.* 2013; Pandruvada *et al.* 2021). One potential explanation for unexplained infertility is anomalies in the sperm centrioles. Sperm centrioles are essential for sperm movement and behavior during swimming and microtubule organization in the early embryo development (Terada *et al.* 2004; Chemes and Alvarez Sedo 2012; Cavazza *et al.* 2021).

It was previously thought that there was only one centriole, the proximal centriole (PC), in mammalian sperm because it was the only recognizable centriole with the canonical barrel shape. Recently it was found that human and cattle sperm have a second, atypical, fan-shaped centriole, the distal centriole (DC) (Fishman *et al.* 2018) ( **Fig 1A-B** ). The distal centriole and proximal centriole form a dynamic basal complex within the sperm neck that mediates the tail beating, generating a head twitching behavior (Khanal *et al.* 2021). In this process, the distal centriole’s left-sided microtubules move up and down relative to the distal centriole’s right-sided microtubules, pushing the rest of the dynamic basal complex. However, the precise contribution of the distal centriole and dynamic basal complex to male infertility is unknown.

Identifying the contribution of sperm centrioles to male infertility depends on identifying appropriate markers and quantifying their relative abundance in the sperm centrioles (Turner *et al.* 2021). These markers can be structural proteins or their posttranslational modifications (Jaiswal *et al.* 2022). Microtubules, composed of the protein tubulin, make up the main structure of the centriole. Post-translational modifications of tubulin can change the overall properties of the microtubule (Janke and Magiera 2020). Microtubules undergo mechanical stress as part of sperm swimming, the distal centriole microtubules, in particular, are under mechanical stress as part of the dynamic basal complex (Khanal *et al.* 2021). Given that tubulin modifications can affect microtubule properties, we wondered if these modifications are present in sperm distal centriole.

Interestingly, the enzymes responsible for the tubulin modifications acetylation, glutamylation, and glycylation are present in the sperm, particularly near the centrioles, suggesting the modifications may be occurring. Acetylation is mediated by acetyltransferase and αTAT1 (Kalebic *et al.* 2013), which are present in sperm (Chawan *et al.* 2020). Recently, it was shown that protein acetylation protects sperm from spontaneous acrosome reactions (Bowker *et al.* 2022). Glutamylation is mediated by glutamylases (TTLL 1, 4, 5, 6, 7, 9, and 11) localized at basal bodies, although TTLL 4, 5, 6, and 7 are also localized to the cilia (van Dijk *et al.* 2007) TTLL1 is found in the sperm and is essential for sperm flagella function (Vogel *et al.* 2010). Tubulin glycylation is mediated by TTLL3 and TTLL8 (Wloga *et al.* 2009; Gadadhar *et al.* 2017). Mouse knockouts of both TTLL3 and TTLL8 have abnormal sperm axonemal dynein activity, flagellar beat, and male fertility (Gadadhar *et al.* 2021). Therefore, tubulin posttranslational modifications may be present in the sperm distal centriole and useful for developing diagnostics and treatments for centriole-based infertility.

Here, we investigated the presence of these modifications in cattle and human sperm by studying their colocalization with known centriolar biomarkers, tubulin and POC1B. As expected, the anti-tubulin and anti-POC1B antibodies labeled two spots in the sperm neck, the distal centriole and proximal centriole, of cattle and humans (Khanal *et al.* 2021) ( **Fig. 1** ). POC1B immunoreactivity is enriched in the distal centriole relative to the proximal centriole (the ratio of distal centriole: proximal centriole, 4.77 ± 2.61, N=101 bovine sperm; 1.24 ± 0.41, N=198 human sperm), this enrichment is more pronounced in bovine than in human. Similarly, Tubulin immunoreactivity is enriched in the proximal centriole relative to the axoneme more pronouncedly in bovine than in human (the ratio of proximal centriole:(proximal centriole + axoneme), 0.91 ± 0.12, N=101 bovine sperm; 0.62 +/- 0.17, N=198 human sperm).

Tubulin is acetylated at lysine residue Lys ^40^ of the a-tubulin subunit (LeDizet and Piperno 1987), which is in the microtubule lumen (Nogales *et al.* 1999; Soppina *et al.* 2012). Acetylation stabilizes microtubules by providing flexibility and resistance to mechanical stress (Portran *et al.* 2017; Xu *et al.* 2017; Eshun-Wilson *et al.* 2019). Tubulin acetylation is reported in centrioles of somatic cells (Sullenberger *et al.* 2020) and sperm (Fishman *et al.* 2018). However, most studies used one antibody that recognizes tubulin acetylation – mouse monoclonal antibody 6-11B-1 (Piperno *et al.* 1987). Here, we tested a second antibody – rabbit monoclonal antibody (Thermo Fisher Scientific, MA533079). Similar to previous studies with antibody 6-11B-1 in sperm, we found that MA533079 labeled the neck and throughout the tail of cattle and human sperm ( **Fig. 1C-D** ). Also, like in previous studies with antibody 6-11B-1, we found that in the neck, MA533079 labeled two spots, the sperm centrioles, colocalizing with POC1B in cattle (98% of cells; N=272; 4 independent stainings) and human sperm (100% of cells, N= 96; 3 independent stainings). Tubulin acetylation staining is weaker in the midpiece axoneme compared to the centrioles in cattle (the ratio of proximal centriole:(proximal centriole + axoneme), 0.58±0.18, N=119 sperm) and human sperm (the ratio of proximal centriole:(proximal centriole + axoneme), 0.54±0.19, N=96 sperm; 3 independent stainings).

Tubulin is glutamylated in the C’ terminus at glutamate residue Glu ^445^ of the a-tubulin subunit, which is on the microtubule exterior (Edde *et al.* 1990). Tubulin glutamylation stabilizes microtubules (Tremoleda *et al.* 2003; Hamel *et al.* 2017). Tubulin glutamylation is present in somatic cell centrioles (Bobinnec *et al.* 1998) and the sperm proximal centriole (Fouquet *et al.* 1994); however, its status in sperm distal centriole remains unknown. We found that anti-glutamylated tubulin antibody GT335 labeled the sperm neck consistently and sometimes the tail midpiece or endpiece in humans and consistently in the endpiece in cattle ( **Fig. 1E-F** ). In the neck, anti-glutamylated Tubulin antibody GT335 labeled two spots, colocalizing with POC1B in cattle (81.5% of 146 cells, 4 independent stainings) and human sperm (73% of N=90 sperm total from 3 men). This staining is enriched in the proximal centriole compared to the midpiece axoneme in cattle (the ratio of proximal centriole:(proximal centriole + axoneme), 0.85 ± 0.21, N=136 sperm) and in human (the ratio of proximal centriole:(proximal centriole + axoneme), 0.56 ± 0.18, N=90 sperm).

Tubulin glycylation is found at glutamate residues Glu ^437^ , Glu ^438^ , Glu ^439^ , and Glu ^441^ in the β-tubulin subunit and glutamate residues Glu ^445^ , Glu ^446^ , and Glu ^448^ in the α-tubulin (Redeker *et al.* 1994) which are all found in the microtubule exterior (Magiera and Janke 2014). The specific effect of tubulin glycylation on microtubules is unclear, but it is strongly suggested that glycylation regulates cilia and flagella length and assembly (Thazhath *et al.* 2004; Gadadhar *et al.* 2021). Tubulin glycylation has not been found in somatic cell centrioles (Gadadhar *et al.* 2017). Glycylation affects sperm flagellar beat (Gadadhar *et al.* 2021), but it was not observed in human sperm proximal centriole using antibody AXO49 mAb and TAP952 mAb, although its presence in the distal centriole remains unknown (Kann *et al.* 1998). We tested two distinct antibodies that label glycylation and found inconsistent labeling. We found that mouse anti-glycylated Tubulin antibody (EMD Millipore, MABS277) diffusely labeled the neck and the tail at the mid-piece and end-piece of cattle sperm (100%, N=19, 3 independent with increasing antibody concentrations) ( **Fig. 1G** ). In human sperm, it did not label the sperm at all (0%, N=10, 3 independent stainings). In contrast, the rabbit anti-glycylated tubulin antibody Adipogen, AG-25B-0034-C100 labeled the cattle sperm neck, midpiece and end piece (94%, N=102, 2 independent experiments). In humans the anti-rabbit glycylated tubulin antibody Adipogen, AG-25B-0034-C100 labeled the midpiece and distal centriole ( **Fig. 1H** ). Midpiece labeling was observed in 100% of cells, and distal centriole labeling was observed in 93.3% of cells (N=15). Overall glycylation in the atypical centriole varies depending on species and antibody and therefore is questionable.

Altogether, our data suggests that acetylation and glutamylation are present in the atypical distal centriole and can be used as sperm centriolar biomarkers in a species-specific manner.

## Methods

Immunofluorescence and quantification was completed as previously described (Turner *et al.* 2021; Jaiswal *et al.* 2022), and the antibody dilutions are described in the reagents section.

## Reagents

Primary Antibodies:

Anti-POC1B made in Mouse (Thermo Fisher Scientific, H00282809-B01P) (Diluted 1 to 300 in cattle and 1 to 100-300 in human sperm). This mouse polyclonal antibody was raised against a full-length human POC1B (aka WDR51B). The antibody specificity was demonstrated by the absence of immunoreactivity in the sperm centriole of rabbits with a POC1B mutation but was immunoreactive in the sperm centriole of control rabbits in our lab.

Anti-POC1B made in Rabbit (Thermo Fisher Scientific, PA5-24495) (Diluted 1 to 100 in cattle and human sperm). The specificity of the antibody was demonstrated in multiples studies showing specific labeling of the centriole (Chang *et al.* 2016; Liu *et al.* 2020; Amargant *et al.* 2021).

Anti-Tubulin made in Sheep (Cytoskeleton, Inc., ATN02) (Diluted 1 to 600 in cattle and 1 to 300 up to 600 in human sperm). The specificity of the antibody toward microtubules was demonstrated in reference (Piroli *et al.* 2014; Lobert *et al.* 2022).

Anti-Acetylated Tubulin made in Rabbit (Thermo Fisher Scientific, MA5-33079) (Diluted 1 to 100 in cattle and human sperm). The specificity of the antibody was demonstrated by labeling purified Chlamydomoanas reinhardtii centrioles (Mahecic *et al.* 2020).

Anti-Polyglutamylated Tubulin made in Mouse (Adipogen, AG-20B-0020-C100) (Diluted 1 to 50 up to 100 in cattle and 1 to 100 in human sperm). The specificity of the antibody was demonstrated by attenuating the activity of TTLL4, the enzyme mediating microtubule polyglutamylation (Arnold *et al.* 2020).

Anti-Glycylated Tubulin made in Rabbit (Adipogen, AG-25B-0034-C100, aka Gly-pep1) (Diluted 1 to 25 up to 100 in cattle and 1 to 100 in human sperm). The specificity of the antibody was demonstrated by attenuating the activity of TTLL3 and TTLL8 the enzymes mediating microtubule monoglycylation (Gadadhar *et al.* 2017).

Anti-Monoglycylated Tubulin made in Mouse (EMD Millipore, MABS277, aka TAP952) (Diluted 1 to 25 up to 100 in cattle and 1 to 100 in human sperm). The specificity of the antibody was demonstrated by attenuating the activity of TTLL3 the enzyme mediating microtubule monoglycylation (Wloga *et al.* 2009; Gadadhar *et al.* 2017).

Secondary Antibodies:

Anti-Sheep Alexa 555 made in Donkey (Thermo Fisher Scientific, A-21436) (Diluted 1 to 1000)

Anti-Mouse DyLight 488 made in Donkey (Thermo Fisher Scientific, SA5-10166) (Diluted 1 to 400)

Anti-Rabbit DyLight 650 made in Donkey (Thermo Fisher Scientific, SA5-10041) (Diluted 1 to 400)

Solutions:

Washing Solution: PBS

Permeabilization Solution: PBS with 0.3% Triton X-100 (PBST) (Sigma Aldrich, 9002-93-1)

Blocking Solution: PBST with 1% BSA (PBSTb) (CHEM-IMPEX INT’L, 00535)

Sperm: Frozen human and cattle sperm were stored in liquid nitrogen and obtained from UToledo andrology clinic and Select Sires Inc. IRB number 202366-UT and 300220-UT, and IBC number108074-UT

Cattle sperm was removed from the straws and human sperm was removed from cryovials. Both types of samples were put onto slides in 5 steps: 1. Straw or cryovials were placed in 37°C water; 2. Sperm was washed using a 40/80 density gradient (Nidacon, Cat # PS40-100 and PS80-100); 3. The pellet was collected and washed in sperm washing media (Nidacon, Cat # PSW-100), then resuspended in M199 (Sigma-Aldrich, Cat # M7528-500ML); 5. Sperm was added to the slide and then dropped into liquid nitrogen where it was stored until use.

Fixation: Methanol (-20°C) (Fisher Chemical, A412P-4)

A humidity chamber is a plastic container that is layered with wet paper towels covered with a lid

Material:

Confocal Microscope: Leica SP8, Sperm images were taken at a magnification of 640× and zoom of 6×, with 512 × 512-pixel density, 3x zoom and 1024x1024-pixel density or 0.75x zoom and 4096x4096-pixel density

Using Photoshop, immunofluorescence sperm panels were cropped to:

- Low magnification: 525 x 1050 pixels and adjusted to 1-inch x 2 inches

- Medium magnification: 150 x 300 pixels and adjusted to 1-inch x 2 inches

- High magnification: 75 x 75 pixels and adjusted to 1-inch x 1 inch.

Adobe Illustrator was used to create sperm images.

Parafilm Wax (VWR, 52858-032)

Cover Slip (VWR, 48366-205)

Slides (Home Science Tools, MS-SLFRO72)

Nail Polish (EMS Diasum, 72180)

Mounting Media made of Fluoroshield with DAPI (Sigma-Aldrich, F6057-20ML)

Hoechst (Thermo Fisher Scientific, H1399) (Diluted 1 to 2000)

LAS X Software Leica using photon counting and BrightR.

Statistical methods: Experiments were completed 2-4 times; we calculated averages and standard deviations using Microsoft Excel.

Primary Antibodies:

Anti-POC1B made in Mouse (Thermo Fisher Scientific, H00282809-B01P) (Diluted 1 to 300 in cattle and 1 to 100-300 in human sperm). This mouse polyclonal antibody was raised against a full-length human POC1B (aka WDR51B). The antibody specificity was demonstrated by the absence of immunoreactivity in the sperm centriole of rabbits with a POC1B mutation but was immunoreactive in the sperm centriole of control rabbits in our lab.

Anti-POC1B made in Rabbit (Thermo Fisher Scientific, PA5-24495) (Diluted 1 to 100 in cattle and human sperm). The specificity of the antibody was demonstrated in multiples studies showing specific labeling of the centriole (Chang *et al.* 2016; Liu *et al.* 2020; Amargant *et al.* 2021).

Anti-Tubulin made in Sheep (Cytoskeleton, Inc., ATN02) (Diluted 1 to 600 in cattle and 1 to 300 up to 600 in human sperm). The specificity of the antibody toward microtubules was demonstrated in reference (Piroli *et al.* 2014; Lobert *et al.* 2022).

Anti-Acetylated Tubulin made in Rabbit (Thermo Fisher Scientific, MA5-33079) (Diluted 1 to 100 in cattle and human sperm). The specificity of the antibody was demonstrated by labeling purified Chlamydomoanas reinhardtii centrioles (Mahecic *et al.* 2020).

Anti-Polyglutamylated Tubulin made in Mouse (Adipogen, AG-20B-0020-C100) (Diluted 1 to 50 up to 100 in cattle and 1 to 100 in human sperm). The specificity of the antibody was demonstrated by attenuating the activity of TTLL4, the enzyme mediating microtubule polyglutamylation (Arnold *et al.* 2020).

Anti-Glycylated Tubulin made in Rabbit (Adipogen, AG-25B-0034-C100, aka Gly-pep1) (Diluted 1 to 25 up to 100 in cattle and 1 to 100 in human sperm). The specificity of the antibody was demonstrated by attenuating the activity of TTLL3 and TTLL8 the enzymes mediating microtubule monoglycylation (Gadadhar *et al.* 2017).

Anti-Monoglycylated Tubulin made in Mouse (EMD Millipore, MABS277, aka TAP952) (Diluted 1 to 25 up to 100 in cattle and 1 to 100 in human sperm). The specificity of the antibody was demonstrated by attenuating the activity of TTLL3 the enzyme mediating microtubule monoglycylation (Wloga *et al.* 2009; Gadadhar *et al.* 2017).

Secondary Antibodies:

Anti-Sheep Alexa 555 made in Donkey (Thermo Fisher Scientific, A-21436) (Diluted 1 to 1000)

Anti-Mouse DyLight 488 made in Donkey (Thermo Fisher Scientific, SA5-10166) (Diluted 1 to 400)

Anti-Rabbit DyLight 650 made in Donkey (Thermo Fisher Scientific, SA5-10041) (Diluted 1 to 400)

Solutions:

Washing Solution: PBS

Permeabilization Solution: PBS with 0.3% Triton X-100 (PBST) (Sigma Aldrich, 9002-93-1)

Blocking Solution: PBST with 1% BSA (PBSTb) (CHEM-IMPEX INT’L, 00535)

Sperm: Frozen human and cattle sperm were stored in liquid nitrogen and obtained from UToledo andrology clinic and Select Sires Inc. IRB number 202366-UT and 300220-UT, and IBC number108074-UT

Cattle sperm was removed from the straws and human sperm was removed from cryovials. Both types of samples were put onto slides in 5 steps: 1. Straw or cryovials were placed in 37°C water; 2. Sperm was washed using a 40/80 density gradient (Nidacon, Cat # PS40-100 and PS80-100); 3. The pellet was collected and washed in sperm washing media (Nidacon, Cat # PSW-100), then resuspended in M199 (Sigma-Aldrich, Cat # M7528-500ML); 5. Sperm was added to the slide and then dropped into liquid nitrogen where it was stored until use.

Fixation: Methanol (-20°C) (Fisher Chemical, A412P-4)

A humidity chamber is a plastic container that is layered with wet paper towels covered with a lid

Material:

Confocal Microscope: Leica SP8, Sperm images were taken at a magnification of 640× and zoom of 6×, with 512 × 512-pixel density, 3x zoom and 1024x1024-pixel density or 0.75x zoom and 4096x4096-pixel density

Using Photoshop, immunofluorescence sperm panels were cropped to:

- Low magnification: 525 x 1050 pixels and adjusted to 1-inch x 2 inches

- Medium magnification: 150 x 300 pixels and adjusted to 1-inch x 2 inches

- High magnification: 75 x 75 pixels and adjusted to 1-inch x 1 inch.

Adobe Illustrator was used to create sperm images.

Parafilm Wax (VWR, 52858-032)

Cover Slip (VWR, 48366-205)

Slides (Home Science Tools, MS-SLFRO72)

Nail Polish (EMS Diasum, 72180)

Mounting Media made of Fluoroshield with DAPI (Sigma-Aldrich, F6057-20ML)

Hoechst (Thermo Fisher Scientific, H1399) (Diluted 1 to 2000)

LAS X Software Leica using photon counting and BrightR.

Statistical methods: Experiments were completed 2-4 times; we calculated averages and standard deviations using Microsoft Excel.
